# Antihypertensive efficacy of the angiotensin receptor blocker azilsartan medoxomil compared with the angiotensin-converting enzyme inhibitor ramipril

**DOI:** 10.1038/jhh.2013.6

**Published:** 2013-03-21

**Authors:** G Bönner, G L Bakris, D Sica, M A Weber, W B White, A Perez, C Cao, A Handley, S Kupfer

**Affiliations:** 1 Park-Klinikum Bad Krozingen, Bad Krozingen, Germany; 2 ASH Comprehensive Hypertension Center, The University of Chicago Medicine, Chicago, IL, USA; 3 Division of Nephrology, Virginia Commonwealth University Health System, Richmond, VA, USA; 4 Division of Cardiovascular Medicine, SUNY Downstate College of Medicine, Brooklyn, NY, USA; 5 Calhoun Cardiology Center, The University of Connecticut School of Medicine, Farmington, CT, USA; 6 Department of Clinical Science, Takeda Global Research and Development, Deerfield, IL, USA; 7 Department of Statistics, Takeda Global Research and Development, Deerfield, IL, USA; 8 Department of Pharmaceutical Development Division, Takeda Pharmaceuticals International, Deerfield, IL, USA

**Keywords:** ACE inhibitor, angiotensin receptor blocker, azilsartan medoxomil, drug therapy

## Abstract

Drug therapy often fails to control hypertension. Azilsartan medoxomil (AZL-M) is a newly developed angiotensin II receptor blocker with high efficacy and good tolerability. This double-blind, controlled, randomised trial compared its antihypertensive efficacy and safety vs the angiotensin-converting enzyme inhibitor ramipril (RAM) in patients with clinic systolic blood pressure (SBP) 150–180 mm Hg. Patients were randomised (*n*=884) to 20 mg AZL-M or 2.5 mg RAM once daily for 2 weeks, then force-titrated to 40 or 80 mg AZL-M or 10 mg RAM for 22 weeks. The primary endpoint was change in trough, seated, clinic SBP. Mean patient age was 57±11 years, 52.4% were male, 99.5% were Caucasian. Mean baseline BP was 161.1±7.9/94.9±9.0 mm Hg. Clinic SBP decreased by 20.6±0.95 and 21.2±0.95 mm Hg with AZL-M 40 and 80 mg vs12.2±0.95 mm Hg with RAM (*P*<0.001 for both AZL-M doses). Adverse events leading to discontinuation were less frequent with AZL-M 40 and 80 mg (2.4% and 3.1%, respectively) than with RAM (4.8%). These data demonstrated that treatment of stage 1–2 hypertension with AZL-M was more effective than RAM and better tolerated.

## Introduction

Hypertension is one of the most important cardiovascular risk factors and its prevalence is still high (Europe 44.2%, North America 27.6%).^1^ When uncontrolled, hypertension is associated with increased risk of myocardial infarction, stroke, general atherosclerosis, dementia and renal failure. High BP is the leading global risk factor for mortality in the world, and the number of attributable deaths worldwide has been reported in the range of 7.5 million cases yearly.^2^ Successful treatment of hypertension is followed by significant reduction in the incidence of comorbid disease and death.^3, 4, 5^ International guidelines or statements for treatment of hypertension^2, 6, 7, 8^ recommend a target BP of <140/90 mm Hg. Despite the availability of numerous pharmacological treatments and guidance for beneficial lifestyle modification, hypertension remains inadequately controlled: In EUROASPIRE III, only about one-third of patients continue to maintain control successfully.^9^

Although there are many effective antihypertensive drugs available today, most are associated with dose-limiting side effects that preclude their use at the higher doses that may be necessary for optimal BP reduction. For example, angiotensin-converting enzyme (ACE) inhibitors are very effective at lowering BP by inhibition of the renin–angiotensin–aldosterone system (RAAS); however, these agents are often associated with significant cough and more rarely with angioedema.^10^ To achieve better BP control and to improve patient adherence with the treatment, it is necessary to prescribe more potent, yet well-tolerated antihypertensive agents. As a class, angiotensin II-receptor blockers (ARBs) have similar or greater efficacy compared with other classes of hypertensive agents but are much more tolerable.^11, 12, 13^ The epidemiological data demonstrate that in spite of the available potent drugs, there is still a need for compounds with improved efficacy for the treatment of hypertension.^9^

Azilsartan medoxomil (AZL-M) is a prodrug that is rapidly hydrolyzed in the gastrointestinal tract during absorption to azilsartan, which has high affinity for the angiotensin II type 1 receptor.^14^ Azilsartan has an estimated bioavailability of 60%, which is not affected by food, and an elimination half-life of approximately 11 h. No drug interactions have been observed in studies of AZL-M or azilsartan.^15^ The present study was designed to compare the efficacy, safety and tolerability of once-daily (QD) AZM-L 40 and 80 mg with QD ramipril (RAM) 10 mg, the most commonly used dose strength and the highest dose approved in Europe.

## Materials and Methods

### Ethical consideration

This study was performed in accordance with the principles of International Conference on Harmonisation/Good Clinical Practice, the Declaration of Helsinki, the current CPMP ‘Notes for Guidance on Clinical Investigation of Medical Products in the Treatment of Hypertension' and all national and country-specific legal requirements. The study protocol was approved by all relevant ethics committees before enrollment of patients. The patient's written informed consent was required before the start of study-related procedures. Patients currently taking antihypertensive drugs had to be willing to discontinue these drugs at screening. The study is registered at ClinicalTrials.gov with the registry number NCT00760214.

### Study design

This phase 3 study was a randomised, double-blind, multicenter trial designed to evaluate the efficacy and safety of QD AZL-M 40 and 80 mg, compared with QD RAM 10 mg, in patients with stage 1 or 2 hypertension after 24 weeks of treatment. Qualifying subjects underwent a 3- to 4-week wash-out period of their former antihypertensive drugs, which coincided with a 2-week single-blind placebo run-in period, 24 weeks of double-blind treatment, and 1 week for follow-up. Eligible patients were randomised in a double-blind manner to one of the three treatment groups: AZL-M 20 mg QD force-titrated to 40 mg QD after 2 weeks, AZL-M 20 mg QD force-titrated to 80 mg QD after 2 weeks, or RAM 2.5 mg QD force-titrated to 10 mg QD after 2 weeks, with continued treatment for an additional 22 weeks. Patients were evaluated for efficacy and safety endpoints at baseline and at weeks 2, 4, 8, 12, 16, 20 and 24 after randomisation. Trough, seated, clinic systolic and diastolic blood pressure (SBP and DBP) were assessed at each visit. Ambulatory blood pressure monitoring (ABPM) was performed at baseline and at the end of week 24. Data at the 1-week follow-up were generated by telephone call.

### Patients

Men and women⩾18 years of age with hypertension were included if their clinic SBP was between 150 and 180 mm Hg inclusive at randomisation and their clinical laboratory profile was not considered clinically significant. Exclusion criteria included: clinic SBP>180 mm Hg or DBP >114 mm Hg at randomisation; secondary hypertension; severe renal disease (estimated glomerular filtration rate <30 ml min^−1^ per 1.73 m^2^); recent history (within 6 months) of a major cardiovascular event or intervention; significant cardiac conduction defects; aortic valve stenosis; use of antihypertensive medications or other concomitant medications known to affect BP; previous history of cancer not in remission for at least 5 years; type 1 or poorly controlled type 2 diabetes mellitus (hemoglobin A1c >8.0%); hyperkalemia (serum potassium >upper limit of normal, 5.5 mmol l^−1^); and night shift work. Pregnant or nursing women and woman of child-bearing potential not using approved means of contraception were also excluded.

### Procedures

Clinic BP measurements were made in triplicate in the nondominant arm after the patient was seated for 5 min using a semiautomated digital BP recorder (Omron HEM 705-CP, Vernon Hills, IL, USA). Every effort was made to ensure that the clinic BP readings were obtained approximately 24 h after the last dose of study medication and before any procedures, including venipuncture.

ABPM was performed on day −1 before randomisation and at the end of week 24, using the Spacelabs Medical Model 90207 (Spacelabs Healthcare, Issaquah WA, USA). During the treatment period, ABPM was initiated immediately after administration of study medication; BP was measured every 15 min during the day (beginning between 0600 and 2200 hours) and every 20 min during the night (between 2200 and 0600 hours).^16, 17, 18^ Quality criteria used for an acceptable ambulatory BP recording included (a) monitoring period ⩾24 h in duration, (b) minimum of 80% of the BP readings expected during the 24-hour period, (c) no more than two non-consecutive hours with less than one valid BP reading, and (d) no consecutive hours with less than one valid BP reading.

Safety assessments included physical examination findings, vital signs and weight, adverse events, clinical laboratory tests and electrocardiographic data. Laboratory parameters were analyzed at a central laboratory. Tolerability and safety were assessed by recording adverse events at all visits. An adverse event was defined as the development of an undesirable medical condition or a deterioration of a pre-existing medical condition. A serious adverse event was an adverse event that resulted in death, was immediately life-threatening, required hospitalisation, resulted in persistent disability, jeopardised the patient or required medical intervention.

### Statistical methods

The primary endpoint was the change from baseline to week 24 in trough, seated, clinic SBP. The primary analysis was an analysis of covariance model for change from baseline to week 24 for clinic SBP. The model included treatment as a fixed effect and baseline clinic SBP as covariate; mean treatment effects and treatment differences (including *P*-values and two-sided 95% confidence intervals) were obtained from the framework of the analysis of covariance model. The type 1 error of 0.05 was controlled using a sequential stepwise procedure that required meeting the statistical objective of each step in order to advance to the next step with a non-inferiority margin of 1.5 mm Hg: step (1) test for noninferiority TAK-491 80 mg vs RAM; step (2) test of significance TAK-491 80 mg vs RAM; step (3) test for noninferiority TAK-491 40 mg vs RAM; and step (4) test of significance TAK-491 40 mg vs RAM. Similar inferential statistical methods were applied to the secondary endpoints. Secondary endpoints included change from baseline to week 24 in trough, seated, clinic DBP, measures of ambulatory BP, and BP response rates (defined as the proportion of subjects who achieved (1) clinic SBP<140 mm Hg and/or a reduction of ⩾20 mm Hg from baseline, (2) clinic DBP<90 mm Hg and/or a reduction of⩾10 mm Hg from baseline, or both (1) and (2)). Safety parameters were summarized using descriptive statistics.

All randomised subjects were included in the analysis of the primary and secondary endpoints (intent to treat), provided subjects had both a baseline and at least 1 post-baseline value. Missing data for the primary and secondary endpoints were handled using last observation carried forward methodology.

A sample size of 270 subjects per group was determined to have at least 90% power to detect a difference of 4.75 mm Hg between the AZL-M and RAM groups by a two-sample *t*-test of the mean change from baseline in SBP, with a 0.05 two-sided significance level, assuming a 14.5 mm Hg s.d. and a 20% dropout rate.

## Results

### Patients

A total of 1229 patients were screened at 106 sites in Europe and Russia, and 1089 patients entered the single-blind period. Of these 1089 patients, 884 met the entry criteria and were randomised to 1 of 3 treatment arms: 295 patients to AZL-M 40 mg, 294 patients to AZL-M 80 mg and 295 patients to RAM 10 mg. A total of 784 of the 884 randomised patients completed the 24 weeks of treatment with double-blind study medication: 265(89.8%) in the AZL-M 40 mg group, 264 (89.8%) in the AZL-M 80 mg group and 255 (86.1%) in the RAM group. The demographics and the baseline characteristics of study population are given in Table 1. There was no significant difference in any parameters. Nearly half of the subjects were male. The mean age was 56.9±11.1 years, and mean body mass index was 29.5±4.7 kg m^−2^. All but four subjects were Caucasian.

Mean baseline clinic and ambulatory measures of SBP and DBP were similar in the three groups (Table 1). Medical history did not differ among the groups. Type 2 diabetes mellitus was reported for 7.5–12.6% of the subjects, and 11.1% of the subjects among the treatment groups reported a significant, ongoing cardiac condition. The most common cardiac conditions were coronary artery disease, angina pectoris and myocardial ischemia.

Antihypertensive agents were the most common previous medications: 47.7% of patients were taking RAAS inhibitors, 19.2% beta blockers, 14.2% diuretics and 13.0% calcium channel blockers. Baseline concomitant medication use was not different among the three treatment groups; the most common were lipid-modifying agents (15.1%), acetylsalicylic acid (14.1%), antidiabetic drugs (6.4%), drugs for gastric acid-related disorders (5.3%) and thyroid therapy (5.3%).

### Clinic BP

**Figure 1:**
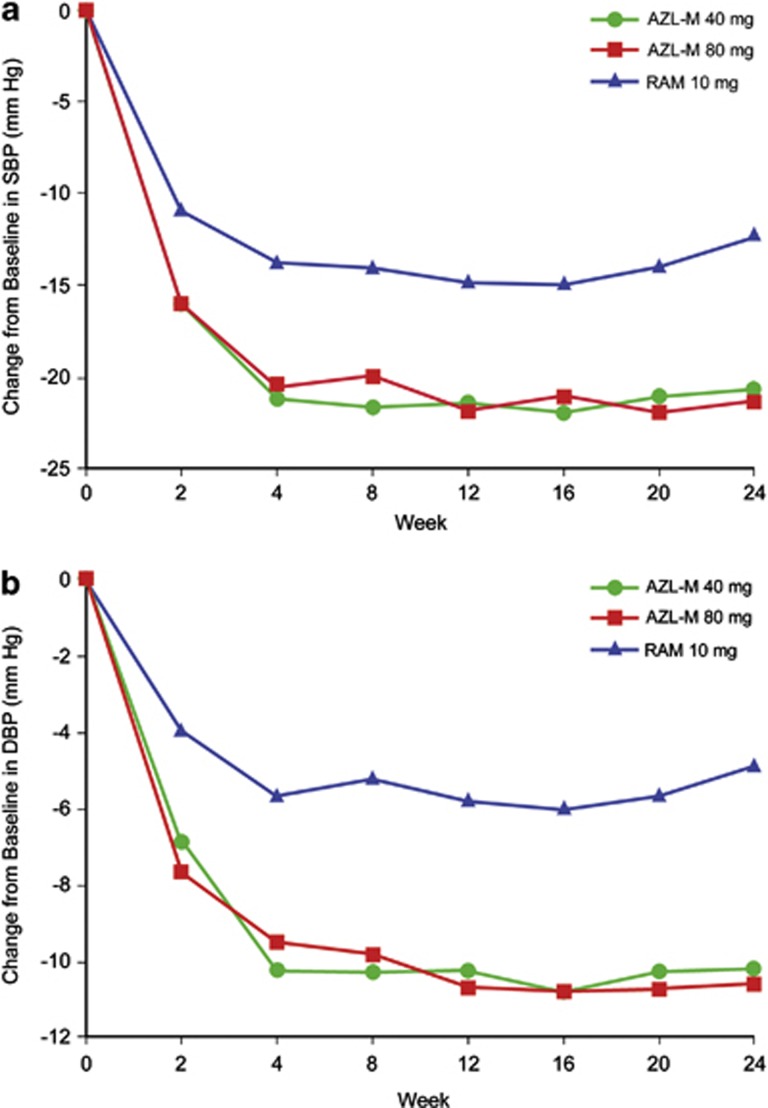
Changes in clinic (**a**) SBP and (**b**) DBP over time. The differences between the two AZL-M groups and the RAM group at week 24 were highly significant for both SBP and DBP (*P*<0.001).

After 24 weeks of treatment, trough, sitting, clinic SBP decreased significantly in all the groups (Table 2). The changes from baseline were significantly greater for the AZL-M 40 and 80 mg treatment groups (−20.6±0.95 and −21.2±0.95 mm Hg, respectively) than for RAM 10 mg (−12.2±0.95 mm Hg) (Table 2). The differences between the AZL-M-treated subjects and the RAM-treated subjects were −8.4 mm Hg for AZL-M 40 and −9.0 mm Hg for AZL-M 80 (*P*<0.001 for both comparisons). Change in trough, sitting, DBP was −10.2±0.55 mm Hg in the AZL-M 40 mg group, −10.5±0.55 mm Hg in the AZL-M 80 mg and −4.9±0.56 mm Hg in the RAM 10 mg group (Table 2). The differences in DBP between the AZL-M-and the RAM-treated subjects were −5.3 mm Hg for AZL-M 40 and −5.7 mm Hg for AZL-M 80, respectively (both *P*<0.001). The majority of the reduction in SBP and DBP was achieved by week 4 after only 2 weeks at the highest dose in each treatment arm and remained nearly unchanged through the end of treatment at week 24 (Figure 1).

### Ambulatory BP

**Figure 2:**
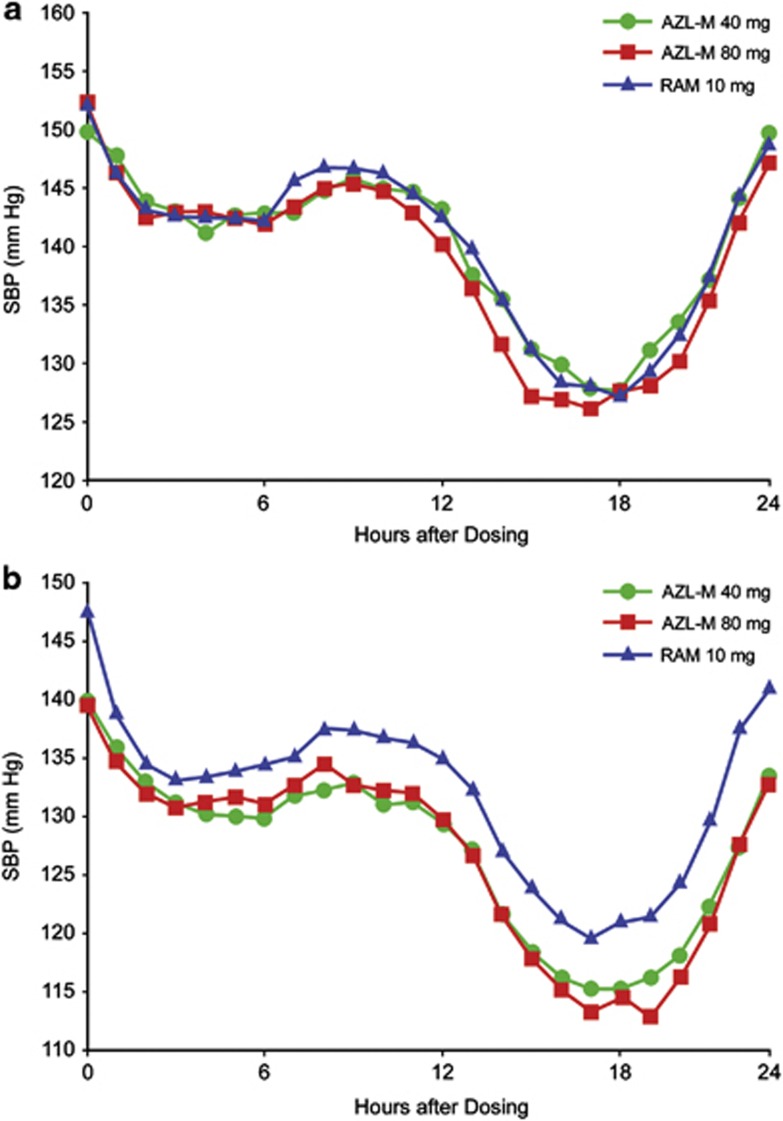
Hourly SBP at (**a**) baseline and (**b**) after 24 weeks of treatment with AZL-M 40 or 80 mg or RAM 10 mg.

Table 2 also summarizes the baseline and change from baseline to week 24 in ambulatory measures of SBP and DBP. AZL-M 40 and 80 mg reduced ambulatory SBP and DBP significantly more than RAM for all ABPM time intervals evaluated, including 24-hour mean, mean daytime, mean nighttime and mean trough pressure. The hourly reductions in ambulatory measures of SBP at (a) baseline and (b) final visit/week 24 are displayed in Figure 2. AZL-M 40 and 80 mg lowered ambulatory SBP to a greater extent than RAM 10 mg at every hour of the 24-h dosing interval.

### Response rates

The proportion of subjects achieving SBP and DBP response criteria is shown in Table 3. The differences between the AZL-M and RAM groups were highly significant (*P*<0.001). More subjects achieved a reduction in clinic BP to <140/90 mm Hg and/or a reduction in BP⩾20/10 mm Hg at week 24 following treatment with AZL-M compared with RAM (54.0% and 53.6% for AZL-M 40 and 80 mg vs 33.8% with RAM 10 mg, respectively; *P*<0.001).

### Subgroup analyses

**Figure 3:**
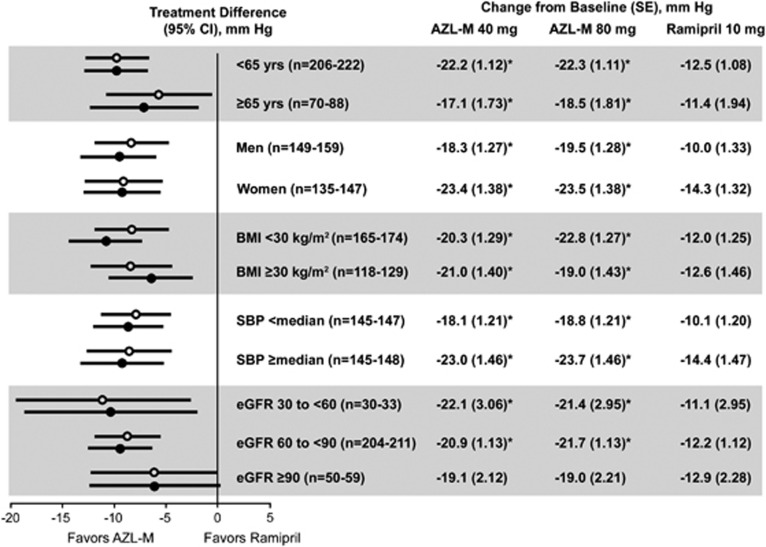
Open circles are seated, clinic SBP treatment differences between the group that received AZL-M 40 mg and the RAM group. Closed circles are the treatment differences between the AZL-M 80 mg group and the RAM group. The median clinic SBP at baseline was 160.3 mm Hg. Baseline estimated glomerular filtration rate (eGFR) categories expressed as ml min^−1^ per 1.73 m^2^. *P<0.05 vs RAM. BMI, body mass index.

Consistent with the overall population, subgroup analyses for the baseline clinical covariates of age, gender, body mass index, clinic SBP and estimated glomerular filtration rate demonstrated statistically significantly or numerically greater BP reduction following treatment with AZL-M 40 or 80 mg compared with RAM (Figure 3).

### Tolerability and safety

Of the 884 patients who were randomised, 880 received⩾1 dose of study medication, and 40.1% experienced an adverse event. The incidence of adverse events was similar in the AZL-M 40 group and the RAM 10 group and slightly higher in the AZL-M 80 group; there were no patterns with respect to the type of serious adverse events reported and the dose of AZL-M (Table 4). Higher rates of cough were reported in the RAM 10 group and higher rates of dizziness and hypotension in the AZL-M groups (Table 4). Adverse events leading to discontinuation were less frequent with AZL-M 40 and 80 mg (2.4% and 3.1%) than with RAM (4.8%). There were no deaths in any of the treatment groups.

Clinically significant increases in serum potassium, sodium and uric acid were observed more often during treatment with the AZL-M 40 and 80 mg as compared with RAM, respectively: potassium >6.0 mmol l^−1^, 2.8, 3.8 vs 1.7% sodium >150 mmol l^−1^, 2.8, 2.1 vs 1.0% and uric acid >506 μmol l^−1^ female or >625 μmol l^−1^ male, 4.1, 3.5 vs 0.7%. The frequency of consecutive creatinine elevations ⩾30% from baseline and greater than the upper limit of normal was low in all the groups: 0.7% and 0.3% for AZL-M 40 and 80 mg, respectively, and none for RAM 10 mg. No subjects had consecutive increases in serum creatinine ⩾50% above baseline and above the upper limit of normal or persistent increases in serum creatinine following discontinuation of study drug.

## Discussion

In antihypertensive treatment, the efficacy and safety of renin-angiotensin system blockade by ACE inhibitors or ARBs is well established. Drugs that inhibit the biological activity of angiotensin II elicit potent BP reductions, are highly protective against end organ damage and may have beneficial metabolic effects, such as delaying the onset of type 2 diabetes.^19, 20, 21^ In clinical studies like the HOPE or the LIFE study, treatment with an ACE inhibitor or an ARB significantly reduced the risk for cardiovascular death, myocardial infarction or stroke, as well as the incidence of new onset diabetes.^22, 23, 24, 25, 26^ The ONTARGET study demonstrated that, in high-risk patients with cardiovascular disease or diabetes, an ARB strategy (telmisartan) was equivalent to an ACE inhibitor strategy (RAM) for the reduction in major cardiovascular events and was better tolerated with lower incidence of cough and angioedema.^27, 28^ The excellent tolerability of the ARB class translates into high patient adherence relative to other antihypertensive drug classes.^29, 30^ Nevertheless, to optimize antihypertensive therapy, more effective drugs that do not sacrifice tolerability are needed. AZL-M is a new ARB with superior efficacy within the ARB class^31, 32, 33^ and characterized by placebo-like tolerability. In the current study, AZL-M was compared with RAM on its blood-pressure-lowering efficacy and its safety and tolerability. RAM was selected as the active comparator owing to its well-established efficacy in treating hypertension and reducing cardiovascular risk and target organ damage, in addition to its well-described safety profile, and the 10 mg dose was evaluated because it is the most commonly used and highest approved dose in Europe.

In the three treatment groups investigated, patients with uncomplicated, stage 1 and 2 hypertension were identical in baseline characteristics and comparable with usual hypertensive patients with respect to age, body weight and accompanying diseases. Both doses of AZL-M were superior to RAM in reducing trough, clinic and ambulatory SBP and DBP, although there were no apparent differences between the 40 and the 80 mg doses. This greater efficacy translated into greater BP control and response rates among subjects treated with AZL-M. Larger BP reductions were consistently observed among patients who received AZL-M relative to RAM in each subgroup examined.

The study also served to evaluate the safety and tolerability of AZL-M at doses within the expected therapeutic range, over a treatment period of 6 months and in comparison with the well-characterized antihypertensive agent RAM. The safety profile of AZL-M observed in this study was comparable with that of RAM with less cough and slightly more dizziness and hypotension among patients treated with AZL-M, the latter likely related to the greater BP reductions achieved with AZL-M relative to RAM. Twice as many subjects (*n*=14, 4.8%) in the RAM group compared with the AZL-M 40 mg group (*n*=7, 2.4%) discontinued study medication owing to adverse events, although the absolute incidence was low. Persistent elevations in serum creatinine were uncommon.

In conclusion, the results from this study demonstrate that AZL-M at doses of 40 and 80 mg QD was significantly superior to RAM 10 mg QD in reducing clinic and ambulatory SBP and DBP. A plateau in BP reduction was reached after 4 weeks of treatment and was maintained throughout the 24 weeks of treatment, illustrating the durability of the BP effects of AZL-M. The better efficacy in BP reduction was consistent with the higher responder rates observed for AZL-M compared with RAM. The overall safety profile of AZL-M 40 and 80 mg observed in this study was similar to that of RAM, with fewer discontinuations due to adverse events. The favorable efficacy and safety profile of AZL-M may translate into better persistence during chronic therapy and more patients achieving BP control.


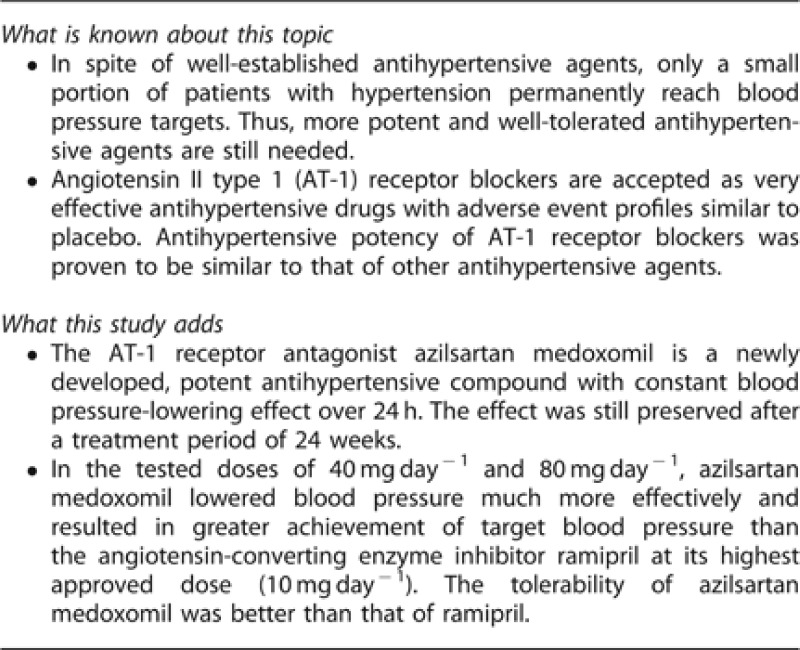


## Figures and Tables

**Table 1:** Demographics and baseline characteristics

*Characteristics*	*AZL-M 40*	*AZL-M 80*	*RAM 10*
Number	295	294	295
Male (%)	53.9	53.7	49.5
Age (years)	56.9±11.5	56.8±11.3	56.8±10.5
BMI (kg m^−2^)	29.6±4.8	29.5±4.7	29.5±4.6
Clinic SBP (mm Hg)	160.7±7.3	161.4±7.7	161.2±8.5
Clinic DBP (mm Hg)	94.7±9.5	95.6±8.7	94.5±8.9
ABPM 24 h mean SBP (mm Hg)	140.7±1.0	139.5±1.0	141.0±1.0
ABPM 24 h mean DBP (mm Hg)	86.4±0.8	86.0±0.7	86.7±0.8
ABPM mean daytime SBP (mm Hg)	143.5±12.7	142.3±13.5	143.4±11.6
ABPM mean daytime DBP (mm Hg)	89.2±9.6	88.0±10.0	88.8±10.1
ABPM mean nighttime SBP (mm Hg)	128.9±14.7	126.4±14.4	128.0±12.8
ABPM mean nighttime DBP (mm Hg)	75.5±11.1	74.0±9.9	74.9±10.7

Abbreviations: ABPM, ambulatory blood pressure monitoring; AZL-M, azilsartan medoxomil; BMI, body mass index; DBP, diastolic blood pressure; RAM, ramipril; SBP, systolic blood pressure.

No statistically significant differences.

**Table 2:** Baseline blood pressure and changes in clinic and ABPM of SBP/DBP after 24 weeks of treatment

*LS mean (s.e.)*	*AZL-M 40*		*AZL-M 80*		*RAM 10*	
	*SBP*	*DBP*	*SBP*	*DBP*	*SBP*	*DBP*
Baseline clinic BP	160.9±0.5	94.8±0.5	161.5±0.5	95.7±0.5	161.4±0.5	94.6±0.5
Change from BL to week 24	−20.6±0.9	−10.2±0.6	−21.2±0.9	−10.5±0.6	−12.2±0.9	−4.9±0.6
						
Baseline 24-h mean ABPM	140.7±1.0	86.4±0.8	139.5±1.0	86.0±0.7	141.0±1.0	86.7±0.8
*Change from BL to week 24*						
24-h mean	−12.7±1.0	−8.0±0.7	−12.3±1.0	−8.3±0.6	−7.8±1.0	−5.3±0.7
Mean daytime (0600–2200 hours)	−12.6±1.0	−8.2±0.7	−12.4±1.0	−8.5±0.7	−8.1±1.1	−5.6±0.7
Mean nighttime (0000–0600 hours)	−12.8±1.1	−7.4±0.8	−12.7±1.1	−8.2±0.8	−6.9±1.1	−4.4±0.8
Mean trough (22–24 h)	−15.6±1.2	−10.2±0.9	−14.9±1.2	−9.9±0.9	−6.7±1.2	−4.5±0.9

Abbreviations: ABPM, ambulatory blood pressure monitoring; AZL-M, azilsartan medoxomil; BL, baseline; DBP, diastolic blood pressure; LS, least square; RAM, ramipril; SBP, systolic blood pressure.

AZL-M vs RAM: *P*<0.05 for all comparisons.

**Table 3:** Response rates after 24 weeks of treatment

	*AZL-M 40*	*AZL-M 80*	*RAM 10*
Subjects, *n*	291	289	290
SBP responders^a^, *n* (%)	174 (59.8)*	166 (57.4)*	113 (39.0)
DBP responders^b^, *n* (%)	220 (75.6)*	215 (74.4)*	159 (54.8)
SBP and DBP responders^c^, *n* (%)	157 (54.0)*	155 (53.6)*	98 (33.8)

Abbreviations: AZL-M, azilsartan medoxomil; DBP, diastolic blood pressure; RAM, ramipril; SBP, systolic blood pressure.

*P*-value for SBP, DBP or joint SBP/DBP response criteria is from a logistic regression with treatment as a factor and either baseline clinic SBP or DBP as a covariate.

*P<0.001 vs RAM.

a Responders are defined as subjects who achieved clinic SBP <140 mm Hg or a decrease of ⩾20 mm Hg at week 24.

b Responders are defined as subjects who achieved clinic DBP <90 mm Hg or a decrease from baseline clinic DBP ⩾10 mm Hg at week 24.

c Responders are defined as subjects who achieved both (a) clinic SBP <140 mm Hg and/or a reduction of ⩾20 mm Hg at week 24 and (b) clinic DBP <90 mm Hg and/or a reduction of ⩾10 mm Hg at week 24.

**Table 4:** Adverse events reported in ⩾2% of the subjects in any treatment group

*Adverse event*	*AZL-M 40*	*AZL-M 80*	*RAM 10*
	*N*=*294*	*N*=*293*	*N*=*293*
Any adverse event	112 (38.1)	128 (43.7)	113 (38.6)
Related to study drug^a^	37 (12.6)	39 (13.3)	41 (14.0)
Leading to discontinuation^b^	7 (2.4)	9 (3.1)	14 (4.8)
Serious adverse event	8 (2.7)	12 (4.1)	6 (2.0)
Deaths	0	0	0
*Events in ⩾2*% *of subjects*			
Nasopharyngitis	19 (6.5)	13 (4.4)	17 (5.8)
Headache	12 (4.1)	10 (3.4)	14 (4.8)
Cough	3 (1.0)	4 (1.4)	24 (8.2)
Increase in blood creatine kinase	6 (2.0)	9 (3.1)	4 (1.4)
Dizziness	8 (2.7)	7 (2.4)	4 (1.4)
Back pain	5 (1.7)	11 (3.8)	2 (0.7)
Contusion	7 (2.4)	4 (1.4)	1 (0.3)
Hypotension	4 (1.4)	6 (2.0)	2 (0.7)
Increase in γ-glutamyl transferase	7 (2.4)	1 (0.3)	3 (1.0)

Abbreviations: AZL-M, azilsartan medoxomil; RAM, ramipril.

Data are *n* (%).

a Definitely, probably, or possibly related, as attributed by the investigator.

b Might include temporary drug interruption or permanent discontinuation and includes all subjects who discontinued the study drug at least once.

## Appendix

### List of study centers and principal investigators

Study centers were located in 10 European countries. The number of patients enrolled per country (*n* (%)) was as follows: Bulgaria, 23 (2.6); Estonia, 71 (8.0); Finland, 16 (1.8); Germany, 183 (20.7); Netherlands, 53 (6.0); Poland, 88 (10.0); Russian Federation, 252 (28.5); Serbia and Montenegro, 125 (14.1); and Sweden, 73 (8.3). The principal investigator was **GB**, Median Klinik, Bad Krozingen, Germany. Members of the multicentre study group (principal investigator at each site in bold) are: **Ingrid Alt**, Vee Family Doctors Center OY, Paide, Estonia; **Viera Ambrovicova**, Eva Bitarovska, CELL B, s.r.o. Interna ambulancia, Levice, Slovak Republic; **Kaja Arbeiter**, Tiia Ruuval, Mirjam Türkson, Pirita Family Doctors Center, Tallinn, Estonia; **Alexander G. Avtandilov**, Galina A. Dudenkova, Nadezda H. Gabitova, Dariya P. Kotova, Nataliya N. Nikitina, Alena A. Pukhaeva, ‘City Clinical Hospital #81' Moscow, Russia; **Alexander Balyabin**, Alla Fomichova, Sergey Sayganov, Valentina Semakova, Irina Tutik, Irina Torosova, MD ‘Pokrovskaya City Hospital', St-Petersburg, Russia; **Boris Y. Bart**, Valentina F. Benevskaya, Ekaterina V. Kudina, ‘Diagnostic Clinical Center #1', Moscow, Russia; **Bettina Bergtholdt**, Iris Filler, Christiane Fleige, Senol Guenaltan, Astrid Novy, Emovis GmbH, Berlin Germany; **Katarina Berndtsson-Blom**, Pia Sandfloo, Ladulaas Kliniska Studier Kungsfors Center, Skene, Sweden; **Ivo G.C.M. Bierens**, Sophia A.E.M. van Cleef, Wiegershof 1, Deurne, The Netherlands; **Jaroslaw Blicharz**, Jacek Karasiński, Tomasz Koziol, Poradnia Kardiologiczna Specjalistyczne Centrum Medyczne, Tarnów, Poland; **GB**, Sabine Boenner, Median Klinik, Bad Krozingen, Germany; **Bertil Borgencrantz**, Erik Sandberg, Capio Läkargruppen, Örebro, Sweden; **Milorad Borzanovic**, Petar Otasevic, Nebojsa Tasic, Srdjan Boskovic, Dragana Dinic, Dedinje Cardiovascular Institute, Belgrade, Serbia; **Hisham Bouzo**, Muenchen, Germany; **Claudia Buettner**, Andrei Khariouzov, Ruth Knorr, Anke Meissner, Klinische Forschung Berlin-Mitte, Berlin, Germany; **Cees Buiks**, Paul Buiks, Ewijk, The Netherlands; **Dmitry Yu. Butko** (replaced by Andrey Obrezan), Olga Amelina, Nina Kaplina, Anna Malykhina, Andrey Obrezan, Natalia Shevchenko, Elena Usikova, MEDEM International Clinic & Hospital, St-Peterburg, Russia; **Julia Chevts**, Alla Reymer, Karlsruhe, Germany; **Boryana Georgieva Chompalova**, Desisilava Baeva, Yordanka Stoyanova Koparanova, Georgi Parishev, IVth Internal Department, MHAT Plovdiv, Plovdiv, Bulgaria; **Andreas Colberg**, Klaus Colberg, Bad Segeberg, Germany; **Willibrordus Alphonsus De Backer**, Cuinera Eliane Marije Hoekstra, Rijswijk, The Netherlands; **Tadeusz Derezinski**, Anna Foltynowicz-Panfil, Beata Wąsikowska, NZOZ ‘Esculap', Gniewkowo, Poland; **Katarina Dulkova**, **MD**, Tomas Dulka, Kardiologicka ambulância, Bratislava,Slovak Republic; **Andrey V. Efimov**, Valeria O. Koreneva, Larisa A. Rogova, Alexander A. Shein, Darya B. Zharavina,‘Central Clinical Hospital #1 of Russian Railways Ltd', Moscow, Russia; **Nikolay V. Efimov**, Nataliya S. Baranova, Sergey A. Bondarev, Irina Mishina, Victor B. Shunkov,‘Railway Clinical Hospital',St-Petersburg, Russia; **Anna Evdokimova**, Natalia Churkina, Marina Iozhkina, Elena Kovalenko, Olga Tereshchenko,‘City Clinical Hospital #52', Moscow, Russia; **Markus Faghih**, Rudolf Eiling, Nicole Friedrichs, Martin Zuelke, Essen, Germany; **Tatiana A. Fedorova**, Alexander V. Mamonov, Tatiana I. Sotnikova, ‘City Clinical Hospital named after S. P. Botkin', Moscow, Russia; **Wijnand Leonard Feis**, Hans Prak, Hendricus Gerardus, Marie Mevissen, Harry Mevissen, Pekela, The Netherlands; **H. Ferguson**, Rotterdam, The Netherlands; **Karin Forck-Boedeker**, Ulrike Gerlach, Ulrich Meyer-Pannwitt, Birgit Moeser, Karin Schneider, Regine Spendlerr, pro scientia med, Luebeck, Germany; **Andrey N. Fursov**, Tatiana Makeeva, Ildar Nagaev, Oleg Parinov, Elena Zakharova,‘Main Military Clinical Hospital named after academician N. N. Burdenko at Ministry of Defense of Russian Federation', Moscow, Russia; **Vladimir Gergel**, Viera Kasperova, Katarina Tothova, Nestatna interna ambulancia, Bratislava, Slovak Republic; **Maria G. Glezer**, Tatiana Ivanova, Svetlana Valovyeva, ‘City Clinical Hospital #59', Moscow, Russia; **lvan Gordeev**, Sona Davtyan, Dmitry Kamenskiy, Ksenia Michailova, Oxana Shaydytik, Evgeny Taratukhin, Natalia Teplova,‘City Clinical Hospital #15 named after O. M. Filatov', Moscow, Russia; **Roman Gorenkov**, Pavel Bogomolov, Olga Dvorina, Olga Kuzmina, Maria Matsievich, Julia Veryaskina, ‘Moscow Regional Scientific Research Clinical Institute named after M. F. Vladimirskiy', Moscow, Russia; **Ewa Gross-Tyrkin**, Malgorzata Szymańska Bujniewicz, NZOZ ‘Non Nocere', Gdansk, Poland; **Markolf Hanefeld**, Frank Schaper, Caren Hoffmann, GWT-TUD GmbH, Zentrum fuer Klinische Studien Forschungsbereich Endokrinologie und Stoffwechselkrankheiten, Dresden, Germany; **Göran Hedberg**, Läkarmottagningen Åkerbäret, Boden, Sweden; **Thomas Hedner**, Lotta Delling, Galina Götherström, Department of Clinical Trials and Entrepreneurship Sahlgrenska Academy at University of Gothenburg Medicinaregatan, Göteborg, Sweden; **Varbitza Todorova Hergeldjieva**, Bohos Bohosyan, Penka Kamenova, Albena Mihaylova, Sadko Pisin, Ognyan Sherbanov, MHAT — Rouse Fourth Internal Cardiology Department, Rouse, Bulgaria; **Claus-Juergen Heydenreich**, Essen, Germany; **Rainer Icén**, Seija Lipponen, Itä-Suomen Lääkärikeskus Oy, Joensuu, Finland; **Stevan Ilic**, Bojan Ilic, Marina Deljanin Ilic, Ljubisa Nikolic, Dejan Petrovic, Institute for treatment and rehabilitation ‘Niska Banja', Niska Banja, Serbia; **Alla Y. Ivleva**, Natalya V. Poluyanova, Olga K. Maksimenko, Elena S. Minina, Tatiyana Selezneva,‘Outpatient Clinic #3', Moscow, Russia; **Dariusz Jastrzębski**, Marcin Chróśielewski, Joanna Kulesza, Anna Rykowska, Jacek Stachera, Wojewódzki Szpital Zespolony w Skierniewicach, Oddzial Chorób Wewnętrznych II, Skierniewice, Poland; **Yanina Kalina**, Natalya Popova,‘City Outpatients Clinic #6', Perm, Russia; **Ilkka Matti Kantola**, Essi Roine, Turku University Hospital, Turku, Finland; **Sakari Kekki**, Taija Mustonen, Joensuun Hoitoasema Oy, Joensuu, Finland; **Kaia Kiiroja**, Iris Koort, Telliskivi Family Doctors Center, Tallinn, Estonia; **Christiane Klein**, Joachim Minnich, Gemeinschaftspraxis Dr. Klein und Minnich, Kuenzing, Germany; **Uwe Kleinecke-Pohl**, Christine Kirsch, Gemeinschaftspraxis Dres. med. Uwe Kleinecke-Pohl, Christine Kirsch Antje Benner-Hemsen, Koeln, Germany; **Anne Kork**, (replacement for Tiina Uuetoa), Anu Hedman, Merle Kadarik, Heli Kaljusaar, Sirje Masik, Mari Reimand, Andres Reinold, Tiina Uuetoa, Rein Vahisalu, East Tallinn Central Hospital, Tallinn, Estonia; **Pekka Koskinen**, Per Ekdahl, Center för Läkemedelsprövningar, Malmö, Sweden; **Victor Kostenko**, Nikolay Bokovin, Victoria Ilina, Zhanna Petrova, Farid Tumarov,‘City Out-Patient Clinic #109', St-Petersburg, Russia; **Karl-Heinz Krause**, Nicola Blind, MedicoKIT Institut fuer Arzneimittelpruefungen, Goch, Germany; **Ernst-Ulrich Krohn**, Mike Mueller-Glamann, Hamburg, Germany; **Mirjana Krotin**, Slavica Banicevic, Aleksandra Djokovic, Ruzica Pokrajac, Slavica Radovanovic, Clinical Center Bezanijska Kosa, Zemun, Serbia; **Włodzimierz Kuś**, Agata Wrzesień-Kuś, Indywidualna Specjalistyczna Praktyka Lekarska, Łódź, Poland; **Riin Lanno**, Elina Šelpakova, Merelahe Family Doctor's Center, Tallinn, Estonia; **Reiner Lehmann**, Andrei Khariouzov, Benno Mertens, Liebhild Stratmann, Klinische Forschung Berlin-Buch GmbH, Berlin, Germany; **Helena Levänen**, Jarmo Liukkonen, Marja Siiskonen, Mikkelin Neurologipalvelu, Mikkeli, Finland; **Carl-Johan Lindholm**, Jörgen Thulin, Hjärtmottagningen Capio-Citykliniken, Lund, Sweden; **Sirje Linntam**, Kullike Avikson, Silvi Kukk, Viive Metsallik, OY Mediteri Family Doctors, Tallinn, Estonia; **Peter Loviska**, Jana Litvinova, Lovicor, s.r.o. Kardiocentrum TN, Trencin, Slovak Republic; **Piotr Mąder**, Wojciech Cieślik, Niepubliczny Zakład Opieki Zdrowotnej Praktyka Dentystyczno—Internistyczna, Poradnia, Kamieniec Ząbkowicki, Poland; **Olga Maus**, Oana Elena Foerster, Vera Matthaeus, Tabea-Maria Weigelt, Zentrum fuer Therapiestudien der Innomed Leipzig GmbH, Leipzig, Germany; **Hendrikus Gerardus Marie Mevissen**, Wijnand Leonard Feis, Huisartsenpraktijk Westerhave, Wildervank, The Netherlands; **Valentina Mincheva Mincheva**, Ivan Tomov Gruev, Krasimira Ilieva Ikonomova, Milen Petkov Minchev, Tatyana Georgieva Petrusheva, Vasil Georgiev Stoyanovski, Veska Marinova Tsanova, Clinic of Cardiology and Lipidology, National Multiprofile Transport Hospital ‘Tsar Boris III', Sofia, Bulgaria; **Jan Nociar**, Andrea Banikova, Kardiomed, s.r.o. Kardiologicka ambulancia, Lucenec, Slovak Republic; **Vladislav Novozhenov**, Boris Kuznetsov, Alexander Matveev, Marina Palchenkova, ‘City Clinical Hospital #29 named after N. PI. Bauman', Moscow, Russia; **Jacek Nowak**, Michel Bońkowski, Łukasz Tomasz Kulak, Lidia Nowak, Andrzej Jan Osika, Waldemar Słowiok, Ewa Kazimiera Źółkiewicz, Prywatny Specjalistyczny Gabinet Internistyczny, Libiąz, Poland; **Andrey G. Obrezan** (replacement for Dmitry Yu. Butko), Olga Amelina, Maria Pechatnikova, Natalia Shevchenko, Elena Usikova, MEDEM International Clinic & Hospital, St-Petersburg, Russia; **Milan Pavlovic**, Svetlana Apostolovic, Miodrag Damjanovic, Ruzica Jankovic, Goran Koracevic, Nebojsa Krstic, Sonja Salinger Martinovic, Svetlana Petrovic Nagorni, Danijela Djordjevic Radojkovic, Milena Radosavljevic, Miomir Randjelovic, Teodora Stanojlovic, Miloje Tomasevic, Snezana Ciric Zdravkovic, Clinical Center Nis, Nis, Serbia; **Danuta Pupek-Musialik**, Wiesław Bryl, Maciej Cymerys, Szpital Kliniczny Przemienienia Pańskiego UM im. Karola Marcinkowskiego w Poznaniu, Katedra i Klinika Choró Wewnętrznych, Zaburzeń Metabolicznych i Nadciśienia, Poznań, Poland; **Biljana Putnikovic**, Radosava Cvjetan, Tijana Kalezic, Miloje Marjanovic, Aleksandar Neskovic, Ivan Stankovic, Ivica Vuksanovic, Clinical Center Zemun, Zemun, Serbia; **Kurt Rack**, Augsburg, Germany; **Dimitar Hristov Raev**, Ivaylo Trifonov Mihaylov, Medical Institute Central Clinical Base, Ministry of Interior, Clinic of Cardiology and ICU, Sofia, Bulgaria; **Heikki Rasi**, Annika Koivisto, Suomen Terveystalo c/o Kalmar Industries, Tampere, Finland; **Aslak Rautio**, Jan Hanning, Quintiles-Hermelinen, Luleå, Sweden; **Gisela Rose**, SlottsbergsklinikenVästra Frölunda, Sweden; **Arvo Rosenthal**, Tallinn, Estonia; Ladislav Ruffini, Nestatna kardiologicka ambulancia, Rimavska Sobota, Slovak Republic; **Axel Schaefer**, Mazin Sanuri, Essen, Germany; **Roland J. Schaetzl**, Grossheirath-Rossach, Germany; **Ernest Schell**, Diana Carrillo-Zink, Praxis Dr. Schell, Nuernberg, Germany; **Cornelia Schiewe**, Hamburg, Germany; **Juergen Scholze**, Bozena Rautenberg, Ursula Grell, Charité Campus Mitte CharitéCentrum fuer Innere Medizin und Dermatologie,Medizinische Poliklinik, Berlin, Germany; **Erik-Delf Schulze**, Marita Schulze, Berlin, Germany; **Ryszard Ściborski**, Grazyna Nosek-Baran, Olga Sosulska, Zespół Opieki Zdrowotnej w Oławie Oddział Chorób, Oława, Poland; **Vasily A. Sergeyev**, Elena N. Bukashkina, Irina V. Sergeeva, Nataliya E. Sharkova, Vladimir L Sinopalnikov, Medical Sanitary Unit #47 ‘Glavmosstroy Hospital', Moscow, Russia; **Boris Sidorenko**, Marina Belous, Irina Iosava, Yulia Polunina, Sergey Vasechkin‘Central Clinical Hospital with Out-patients' Clinic of Russian Federation President's Management Department, Moscow, Russia; **Olov Sjöberg**, Göteborg, Sweden; **Konstantin E. Sobolev**, Ayzhan Abildinova, Tatyana Shokina,‘City Clinical Hospital #59', Moscow, Russia; **Mai Soots**, Ester Keba, Foundation Viljandi HospitalViljandi county, Estonia; **Andrey V. Strutynsky**, Egor N. Banzelyuk, Alexey B. Glazunov, Alexey A. Reysner, ‘City Clinical Hospital #31', ‘Russian State Medical University of Roszdrav', Moscow, Russia; **Maciej Strawczyński**, Wojciech Borowski, Piotr Mariusz Drzewek, Romuald Korzeniak, Radosław Marek Lubasiński, Specjalistyczna Przychodnia Lekarska ‘Medikard', Płock, Poland; **Ireneusz Szymczyk**, Alicja Jabłońska, Mariusz Kopeć, Praktyka Lekarzy Rodzinnych ‘Salus', Katowice, Poland; **Sergey Tereschenko**, Pavel Galaktionov, Irina Kositsyna, Tatiana Uskach,‘City Clinical Hospital #68',‘Moscow State Medicine and Dentistry University of Roszdrav', Moscow, Russia; **Maire Treial**, Űlle Roostalu, Maarjavälja Family Doctor's Center, Tartu, Estonia; **Haralin Stoianov Tumbev**, Daniela Alexandrova Tumbeva, MMA-Hospital Base for Active Treatment, Varna, Bulgaria; **Maria Lyubomirova Tzekova**, Nikolay Iliev Iliev, Rayna Petrova Buyuklieva, Clinic of Cardiology and Intensive Care Unit, UMHAT ‘Dr Georgi Stranski' Pleven, Pleven, Bulgaria; **Rudolf Uhliar**, Veria, s.r.o. Kardiologicka ambulancia, Bratislava, Slovak Republic; **Ilie Urlea-Schoen**, SiegResearch Pharma, Siegen, Germany; **Tiina Uuetoa** (replaced by Anne Kork), Anu Hedman, Merle Kadarik, Heli Kaljusaar, Anne Kork, Sirje Masik, Mari Reimand, Andres Reinold, Rein Vahisalu, East Tallinn Central Hospital, Tallinn, Estonia; **Gerardus Johannes Maria van Doesburg**, Hanne Maria van Lange (maiden name Punte), Richard Everhardus Arnoldus Jansen, Lichtenvoorde, The Netherlands; **Viviënne Eleonore Katrien Maria van de Walle**, Beek, The Netherlands; **Harry F.C.M. van Mierlo**, Britta Patricia Tasseron, Karin van Koerten, Roelofarendsveen, The Netherlands; **Zorana Vasiljevic**, Snezana Komnenovic, Jasminka Kostic, Gordana Krljanac, Gordana Matic, Branislav Stefanovic, Clinical Center of Serbia Emergency Hospital–Department of Urgent Cardiology, Belgrade, Serbia; **Monika Vask**, Nõlvaku Family Doctor's Center, Tartu, Estonia; **Yury Vasyuk**, Elena Nesterova, Irina Sadulaeva, Elena Shupenina,‘City Clinical Hospital #33', ‘Moscow State Medicine and Dentistry University of Roszdrav', Moscow, Russia; **Juergen Wachter**, Mannheim, Germany; **Bernhard R. Winkelmann**, Cornelia Gerlinde Finger, Kardio-Fit GmbH & Co. KG, Frankfurt, Germany; **Vladimir Zadionchenko**, Oxana Nesterenko, Alexey Schikota,‘City Clinical Hospital #11', Moscow, Russia; **Peter Zareczky**, Drahomira Bollova, Cardio, s.r.o. Kardiologicka ambulancia, Galanta, Slovak Republic; **Oleg Zharkov**, T.Dogadova, Andrey Gvozdkov, D.Marchak, Lidia Sokolova, Federal State Institution ‘National Medical Surgical Center named after N. I. Pirogov', Moscow, Russia.

## Notes

This study was sponsored by Takeda Global Research and Development Center. There was no payment by the sponsor to any authors for their work in analyzing data or writing or editing of this article. The principal investigator was GB, Park-Klinikum, Bad Krozingen, Germany. GB has a consultant relationship with Takeda Pharma Germany and is a member of the speakers' bureau for Takeda Pharma Germany, Merck Pharma, Germany, Merck, Sharp and Dohme, Boehringer Ingelheim, Daiichi Sankyo and AstraZeneca. DS has had a research and/or consultant relationship with Takeda Pharmaceuticals, Boehringer-Ingelheim, Novartis and Merck. GLB has received grant or research support from Forest Labs, Medtronic and Relypsa and is a consultant for Takeda, Abbott, CVRx, Johnson & Johnson, Eli Lilly and Daiichi Sankyo. He is a member of the speakers' bureau for Takeda. WBW has served as a paid safety consultant for Takeda Global Research and Development Center. MAW is a member of the speakers' bureau and consultant for Boehringer-Ingelheim, Daiichi Sankyo, Forest, Novartis and Takeda; and AP, CC, AH and SK are full-time employees of Takeda Global Research and Development Center (Deerfield, IL, USA).
